# A question of scale: Human migrations writ large and small

**DOI:** 10.1186/1741-7007-8-98

**Published:** 2010-07-21

**Authors:** Murray P Cox, Michael F Hammer

**Affiliations:** 1 Institute of Molecular BioSciences, Allan Wilson Centre for Molecular Ecology and Evolution, and the Bio-Protection Centre, Massey University, Palmerston North, New Zealand; 2 ARL Division of Biotechnology, University of Arizona, Tucson, AZ 85721, USA

## Abstract

Several recent papers illustrate the importance of migration and gene flow in molding the patterns of genetic variation observed in humans today. We place the varied demographic processes covered by these terms into a more general framework, and discuss some of the challenges facing attempts to reconstruct past human mobility and determine its influence on our genetic heritage.

See research articles: http://www.biomedcentral.com/1741-7007/8/15 and http://www.biomedcentral.com/1471-2156/11/18

## 

Migration is a major force shaping patterns of genetic variation in humans [1]. The history of our species is dominated by movement: from the global expansion of modern humans out of Africa, to continent-wide dispersions following the development of agriculture, to the often socially dictated movement of individuals seeking mates or resources. As can be seen from even these simple examples, migration can occur over a variety of spatial and temporal scales - from the wholesale dispersal of populations to the movements of individuals. Large-scale dispersals cause populations to diverge as they subsequently accumulate mutations and experience genetic drift, while individual mobility over smaller geographical scales counteracts these forces by sharing genetic variants between populations. Thus, each of these forms of migration has characteristic effects on human population structure and each brings its own set of methodological challenges [2,3].

A major aim in contemporary molecular anthropology is to determine when gene flow has occurred (that is, temporal control) and over what geographical range its effects were felt (that is, spatial control) (Figure 1). Consider these examples: the expansion of modern humans out of Africa occurred approximately 50,000 years ago and had global impact (circle a); dispersals following the development of agriculture occurred <10,000 years ago, but were largely restricted to continental (or sub-continental) regions (circle b); gene flow driven by intermarriage between adjacent small communities is often of only local effect and frequently occurs over short time scales (circle c).

**Figure 1:**
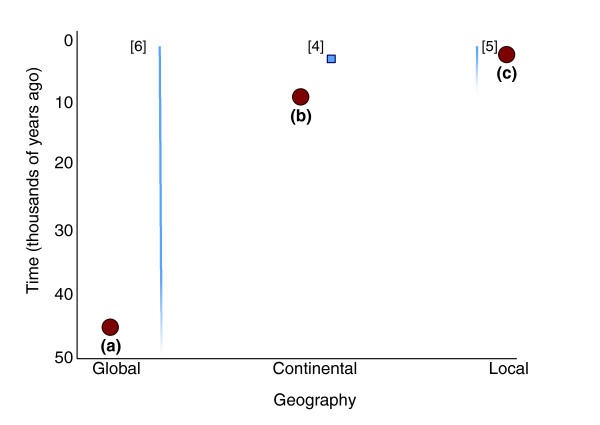
**Time and space are important concepts for characterizing gene flow in human history**. Labels indicate approximate temporal and spatial scales represented by research described in the text. Blue lines illustrate uncertainty about the time of migration, whereas blue boxes represent tighter temporal estimates of gene flow. Red circles represent specific examples of human movement discussed in the text: (a) the expansion of modern humans out of Africa approximately 50,000 years ago (global impact); (b) dispersions following the development of agriculture <10,000 years ago (largely restricted to continental regions); and (c) gene flow driven by intermarriage between adjacent small communities (often recent with only local effects).

In practice, it remains difficult to determine the timing and geographic extent of specific episodes of historical human migration. Spatial control largely reduces to an issue of appropriate sampling (that is, sufficiently representing the full geographical range over which migration occurred). Increased geographical sampling is currently an expensive proposition, but advances in genotyping technologies are likely to reduce experimental costs to more reasonable levels in the near future. Attaining fine temporal control is a far more difficult prospect. To date, tight temporal resolution of human mobility has largely been the sole purview of ancient DNA studies. However, new analytical methods are currently being developed that can place chronological bounds on human gene flow using genetic data from individuals living today. The choice of genetic marker is also a major influence on what researchers can reveal about historical movements. Until recently, mitochondrial DNA (mtDNA) and the Y chromosome were favored as the most informative genetic markers available for studying human history. mtDNA is passed only from a mother to her offspring, while the Y chromosome is passed only from a father to his sons. This simple mode of transmission makes mtDNA and the Y chromosome easy model systems for studying human ancestry. In particular, these loci can be used to detect whether migration has occurred between populations in the past.

## Global connections

The authors of a recent article in *BMC Biology *adopted exactly this approach. Li and colleagues [4] report mitochondrial and Y chromosome data for 23 inhumations at the Xiaohe cemetery in Western China's Tarim Basin. Here, ancient DNA techniques were employed to characterize the genetic diversity of samples fixed in both time and space. The Xiaohe community existed around 4,000 years ago on the ancient east-west network of trade routes known collectively as the Silk Road. Despite straddling the border of East Asia, 'Caucasoid' mummies, artwork influenced by ancient western civilizations and records written in Tocharian, an extinct branch of the Indo-European language family, amply illustrate the region's close connections with the west. Li and colleagues contribute to this extensive evidence of east-west links by announcing the presence of West Eurasian mtDNA and Y chromosome lineages among Xiaohe's dead.

However, mtDNA and Y chromosome records are necessarily biased towards the sex-specific movements of women and men, respectively. Further, demographic reconstructions made from mtDNA and the Y chromosome have considerable uncertainty due to the substantially higher rates of stochastic genetic drift in these uniparentally inherited systems compared to autosomal loci. Ultimately, mtDNA and the Y chromosome are just two small loci in a vast ocean of human genetic variation. In contrast, the autosomes consist of millions of independently evolving regions, any number of which can be surveyed to characterize gene flow in humans. Today, the rapid advent of new genotyping technologies is allowing massive numbers of markers to be screened across the human genome. Xu and colleagues [5] adopted this approach in a recent article in *BMC Genetics*. They report approximately 50,000 single nucleotide polymorphisms (SNPs, or point mutations) drawn from the nuclear genome. These markers were genotyped in individuals from two indigenous communities in Thailand that show linguistic and anthropological evidence of prehistoric connections. Using a suite of clustering methods, Xu and colleagues demonstrate that the Mlabri and Htin share more nuclear variants with each other than either does with surrounding populations.

While determining whether two populations share genetic variation is a relatively simple exercise, identifying and quantifying the amount of gene flow between them requires more advanced modeling and inferential statistics. This is usually applied within a framework of coalescent theory. Large quantities of genetic information are required to infer rates of gene flow, and data sets of this size have only recently become feasible. In a 2008 article in *BMC Genetics*, we adopted exactly this sort of strategy to quantify rates of gene flow on a global scale [6]. Instead of screening many pre-ascertained point mutations like Xu and colleagues, we instead fully sequenced 20 large genomic regions distributed across the human X chromosome in 90 individuals from six globally distributed populations (Mandenka, Biaka and San in Africa, and French Basques, Han Chinese and New Guinea Highlanders in Eurasia). This research was unique because these 20 genomic regions were chosen specifically to be recombinationally unlinked (that is, independent) and selectively neutral (that is, located far away from genes), in marked contrast to most studies where genetic variants are tightly linked to functional sites that are potentially affected by natural selection (for example, mtDNA and the Y chromosome) or even occur within selected loci (for example, SNP chip data where many polymorphisms are located in, or close to, genes). We found that worldwide rates of gene flow (*m*) were approximately five-fold higher among non-African populations relative to African groups [6]. Interestingly though, effective population sizes (*N*) and migration rates (*m*) are inversely proportional in African and Eurasian groups - although migration rates are approximately five-fold lower in Africa, effective population sizes are approximately five-fold higher. Consequently, population migration rates (*Nm*) are globally very similar (*Nm *= 2.4). In other words, we found that approximately two migrants per generation have moved between these globally distributed populations on average through time. Some of these movements may have been recent, despite the large geographical distances between populations, but most would have occurred when these populations were geographically much closer (for example, during the initial expansion of anatomically modern humans out of Africa). Developing statistical methods to determine exactly when this gene flow occurred remains an important outstanding task.

Today, sequencing and comparing entire human genome sequences is the surveying method of choice. For example, the complete genomes of many individuals have now been sequenced, including such famous names as Archbishop Desmond Tutu [7] and Nobel Laureate James Watson [8]. Genome sequences yield orders of magnitude more information than either the SNPs screened by Xu and colleagues, or the small genomic regions (comparatively speaking) sequenced in our 2008 research program.

## Gene flow from Neandertals

**Figure 2:**
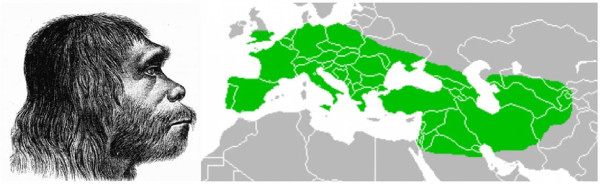
**Illustration of a Neandertal man with a map showing the geographical range of Neandertals as currently understood**. Neandertals lived across Europe from approximately 400,000 to approximately 30,000 years ago.

One recent study has compared entire genomes to provide a surprising new insight into human history. Just this year, Green and colleagues [9] sequenced the complete genome sequence of the Neandertal, a sister group to modern humans that lived in Europe until approximately 30,000 years ago (Figure 2). Until then, only mtDNA sequences were available from Neandertals, largely because mtDNA occurs in multiple copies per cell, which helps circumvent difficulties extracting DNA from ancient remains. These mtDNA sequences suggested that no gene flow had occurred between Neandertals and modern humans, although this absence of evidence was never entirely conclusive because even mtDNA lineages introduced at high frequency could easily have been lost through genetic drift. Using revolutionary new sequencing technologies, Green and colleagues have now read an entire composite genome sequence from three Neandertal individuals. Their study benefits from complete sequence information (that is, sequences rather than SNPs), a large number of independently evolving regions drawn from the nuclear genome, as well as tight spatial and temporal control due to the detailed archaeological record available for their samples. In contrast to the story told by mtDNA, this dataset presents clear evidence of gene flow from Neandertals into modern humans. Humans of non-African descent carry at least 13 large genetic regions that ultimately derive from our Neandertal siblings. Because these genetic regions are not found in people of African descent, this gene flow must have occurred - from Neandertals into modern humans - once some of our ancestors had already left Africa around 50,000 years ago to settle the rest of the world.

Although this particular episode of gene flow has been characterized with good spatial and temporal resolution, similar detail is rare in other studies of human gene flow. Indeed, many questions still remain about how gene flow has helped shape patterns of genetic variation in humans. mtDNA and the Y chromosome are just two small genetic regions within a very large genome, and as the Neandertal study shows, they are not necessarily representative of much of our genetic diversity largely due to their increased rates of genetic drift. Furthermore, sampling ancient DNA becomes increasingly difficult at ever greater time depths - at 4,000 years old, the Xiaohe cohort stretches back less than one-tenth of the continental history of Eurasia, a region first settled after 50,000 years ago. The Neandertal study does reach back this far, but reflects a contact event that was probably over quickly; by approximately 40,000 years ago, modern humans were dominant across Eurasia, and Neandertals were on the wane. For most other questions involving human movements, reconstructing an inclusive spatial and temporal history will depend on the vast genomic datasets now being produced for a wide range of populations. In tandem, improved inference methods must be developed to attain this goal. With the exception of ancient DNA studies, current rates of global gene flow are all long-term estimates (that is, migration rates are averaged over very long time spans). Our inability to resolve the timeframes over which specific demographic processes occurred is a major limitation that requires further attention.

## Adding complexity

Finally, we note that human migration cannot simply be characterized by the timing and geographical extent of gene flow. Human mobility is far more nuanced, and at least four additional axes would seem to warrant further consideration. First, rates of gene flow are hard to determine with certainty. Although the presence (or absence) of migration can often be inferred relatively easily, estimates of actual rates frequently have considerable uncertainty [6]. Methods allowing better resolution of rates of gene flow are required. Second, migration can be sex specific. Although movement into the Xiaohe community was apparently not sex biased [4], sex specific mobility has been observed elsewhere. For instance, the spread of Asian alleles into Island Southeast Asia appears to have favored Asian women over Asian men [10]. Adequate sampling of sex-linked markers (for example, X chromosome *versus *autosomes) is a prerequisite for detecting such patterns. Third, gene flow can be directional (for example, movement along the Silk Road) or non-directional (for example, isolation-by-distance). Directional mobility along the Silk Road will produce quite different genetic patterns than intermarriage between adjacent communities. Improved regional sampling will be necessary to detect such patterns. Fourth, rates of gene flow may change over time. Varying rates of gene flow strongly affect patterns of genetic diversity [6], although to our knowledge, no statistical methods have yet been developed to detect real-world examples of these processes.

**Figure 3:**
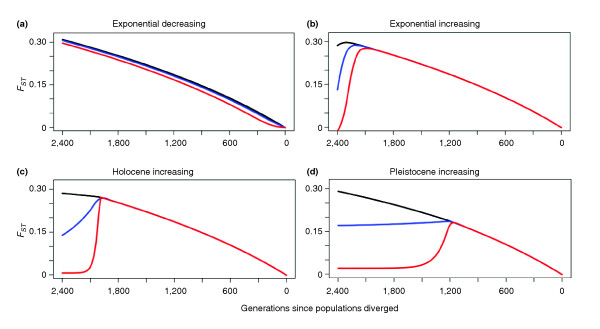
**Population divergence (*F_ST_*) versus changing rates of gene flow (*Nm*) through time**. Panels illustrate the effects of migration rates changing in different ways over time: **(a) **gene flow decreasing exponentially as two populations separate; **(b) **gene flow increasing exponentially as two populations come back into contact; **(c) **sudden increase in gene flow 10,000 years ago in the Holocene; and **(d) **sudden increase in gene flow 30,000 years ago in the Pleistocene. Black, blue and red lines reflect population migration rates (*Nm*) that change through time as described for each of the model scenarios listed above. These lines represent long-term gene flow averages (*Nm*) of 0.033, 0.33 and 3.3, respectively. Importantly, the total amount of gene flow does not differ between lines with the same color.

We expand on just one of these points for illustration (Figure 3). Even when gene flow is inferred explicitly, existing methods invariably assume that it has remained constant through time. However, it seems more reasonable that two diverging populations might share more migrants initially (due to shared geography or existing social relationships), with gene flow subsequently decreasing exponentially as the two populations move apart (Figure 3a). Or gene flow might increase exponentially as two geographically separated populations begin to move closer together (Figure 3b). Alternatively, gene flow might suddenly resume between two long separated populations; for instance, where geographically disconnected populations came back into contact, either as hunter-gatherer groups during the late Pleistocene (Figure 3d), or as human mobility increased following the development of farming in the Holocene (Figure 3c). The important point is this: two populations can look very similar (*F_ST _*= 0) or very different (*F_ST _*= 0.3) even when they have exchanged *the same number of migrants *(that is, graph lines with the same color in figure 3). It is therefore insufficient to consider only how many migrants have moved between populations; we also need to know when these movements occurred. Although these four particular cases are hypothetical, the effects on genetic diversity of changing migration rates through time are real enough. We anticipate that this topic alone will stimulate a productive field of future research.

The series of migration vignettes presented in this commentary illustrates just how little we understand about the role of migration and gene flow in human history. Recent studies have shed some light on the processes affecting movement among communities, between continents and across the globe, but more sophisticated methods are needed. We look forward to meeting these methodological challenges and the clarity that a more nuanced understanding of human mobility will bring.
